# Acute anterior myocardial infarction in a 22-year-old male nephrotic patient along with familial hyperlipidaemia

**DOI:** 10.1017/S1047951118001130

**Published:** 2018-07-24

**Authors:** Junzhong Zeng, Jinhua Li, Jiyu Zhang

**Affiliations:** Department of Cardiovascular Disease, Qingyuan People’s Hospital, Guangzhou Medical University, Qingyuan, Guangdong, China

**Keywords:** Nephrotic syndrome, acute myocardial infarction, familial hyperlipidaemia, case report

## Abstract

We report a case of acute myocardial infarction in a nephrotic male patient. A 22-year-old man with a 1-year history of nephrotic syndrome due to membranous nephropathy presented with acute chest pain and was admitted to our emergency room. An electrocardiogram showed ST elevation in leads consistent with anterior and inferior myocardial infarction. Subsequent cardiac catheterisation showed evidence of thrombotic occlusion of the anterior descending coronary artery. The patient had no long history of hypercholesterolaemia or hypertriglyceridaemia. The case suggests that young patients with a short-term nephrotic syndrome may be at an increased risk for acute coronary syndrome owing to hypercoagulability state.

As the standard of living is becoming higher in China, blood cholesterol and triglyceride levels of the Chinese population are steadily rising gradually.^1^ In spite of the amelioration of life quality and the improvement of prognosis by the emergence and application of antilipaemics such as statins in middle-aged and senior individuals, it is easy to find teenagers with myocardial infarction or nephrotic syndrome who rarely used statins.^2^ However, it is rare to find young adults affected with both diseases at the same time. Once an individual is affected by both diseases, it causes great damage and is sometimes fatal. Here, we report a rare case of nephrotic syndrome associated with acute myocardial infarction in a 22-year-old male patient, and we investigate the diagnosis, treatment, and prevention. We hope to provide experience to deal with patients who suffer from both nephrotic syndrome and acute myocardial infarction in the meantime.

## Case presentation

**Figure 1:**
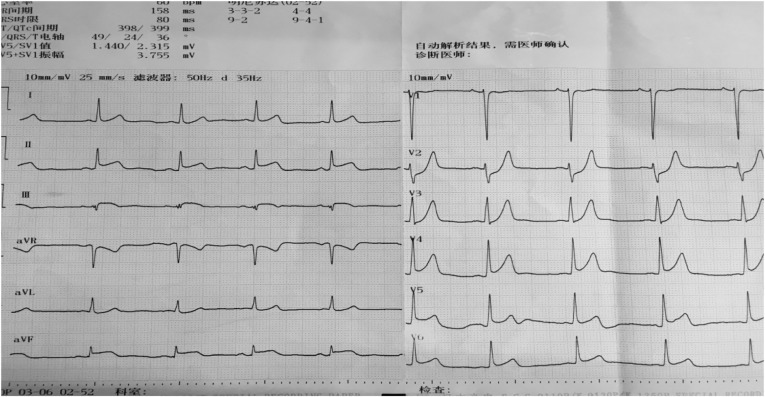
Electrocardiogram on admission shows elevated 0.1–0.2 mv in II, III, aVF, and V4–V6.

A 22-year-old male patient with a past medical history of nephrotic syndrome due to membranous nephropathy, which was diagnosed by renal biopsy 1 year ago, was admitted to the emergency room and transferred to our cardiac care unit with the complaint of chest pain along with dizziness, headache, and radiating pain to his left arm for 4 hours. The patient took no drugs in the past 6 months. On clinical examination, his pain was reported to be 5/10 according to the visual analogue pain scale, he was afebrile, with a maximum temperature of 36.6°C, and had ortho-arteriotony, with a blood pressure of 128/85 mmHg, and no tachycardia or bradycardia (pulse rate of 66 beats per minute). The patient’s face was bloated, but there was no jugular venous distention, muffled breath sounds, rhonchi or moist rale, the third or fourth heart sound gallop, pericardial rub, peritoneal irritation, hepatosplenomegaly, or leg oedema. Electrocardiogram on admission showed that ST elevated to 0.1–0.2 mv in II, III, aVF, and V4–V6 (Fig 1).

Initial abnormal serum laboratory results were as follows: troponin I, 3.499 ug/L; myoglobin, 1200 ug/L; creatine kinase-MB, 69 U/L; creatine kinase, 678 U/L; lactate dehydrogenase, 343 U/L; cholesterol, 6.43 mmol/L; low-density lipoprotein cholesterol, 5.02 mmol/L; glucose, 2.44 mmol/L; high-density lipoprotein cholesterol, 0.76 mmol/L; prothrombin time, 10.3 s; activated partial thromboplastin time, 24.5 seconds; serum total protein, 39.1 g/L; albumin, 20.2 g/L; and 24-hour urine protein, 11.054 g/24 hours.

**Figure 2:**
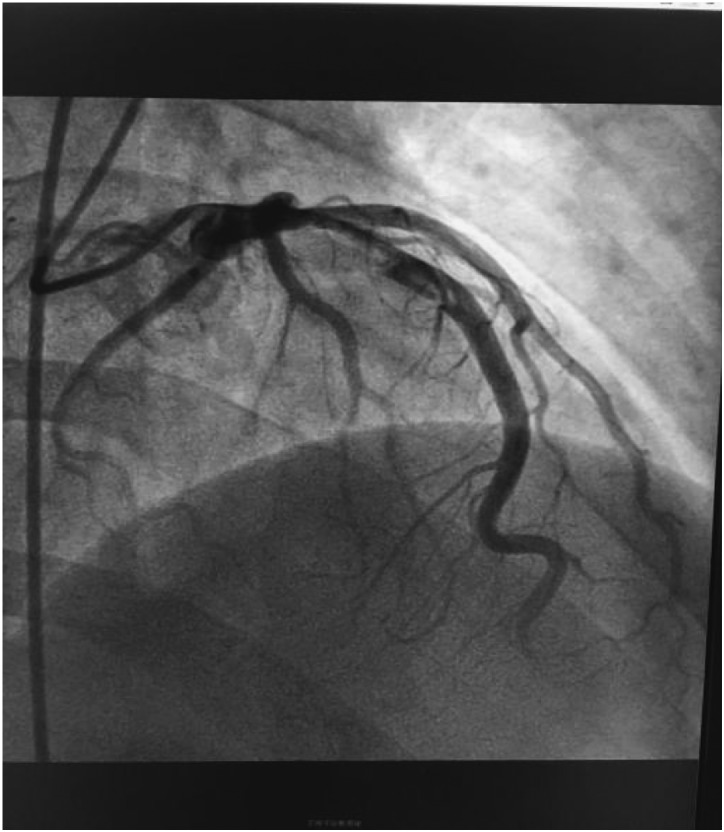
Before the thrombus suction operation.

**Figure 3:**
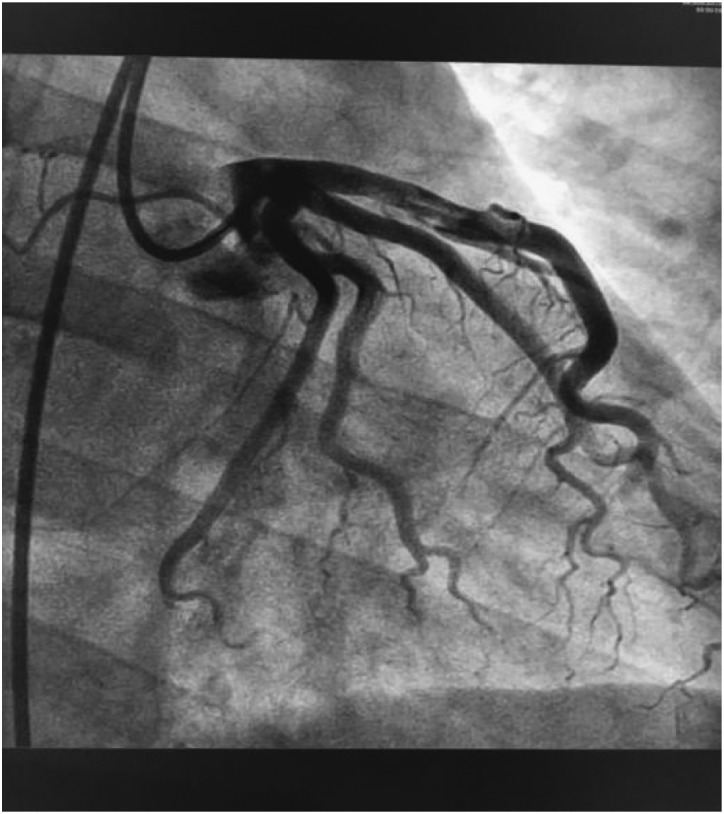
After the thrombus suction operation.

According to the results, our preliminary diagnosis was acute inferior myocardial infarction (Killip grade I) combined with nephrotic syndrome, and we performed a percutaneous intervention procedure, which was followed by 300 mg aspirin and 300 mg clopidogrel orally immediately. During the operation, we saw that the left coronary artery had thickened and the left main artery, left circumflex artery, and right coronary artery were normal. However, there was a great deal of thrombi blocking the anterior and middle anterior descending branch, resulting in 95% stricture (Fig 2) and leading to bloodstream classification at thrombolysis and thrombin inhibition in myocardial infarction grade II. Next, we applied the thrombus suction technique and injected 13 ml of tirofiban to dissolve blood clots. Unfortunately, there was still medium thrombi remaining and 60% stricture in the re-examination of coronary arteriography (Fig 3). The bloodstream classification at thrombolysis and thrombin inhibition in myocardial infarction grade III. Because the anterior descending branch dilatation made it unsuitable for stent implantation, we had to stop further operation. Next, we gave him a therapeutic regimen of low-molecular-weight heparin for 7 days, 100 mg aspirin per day, and 90 mg ticagrelor twice a day to fight platelet aggregation, and 40 mg atorvastatin per night to remedy hyperlipidaemia persistently.

**Figure 4:**
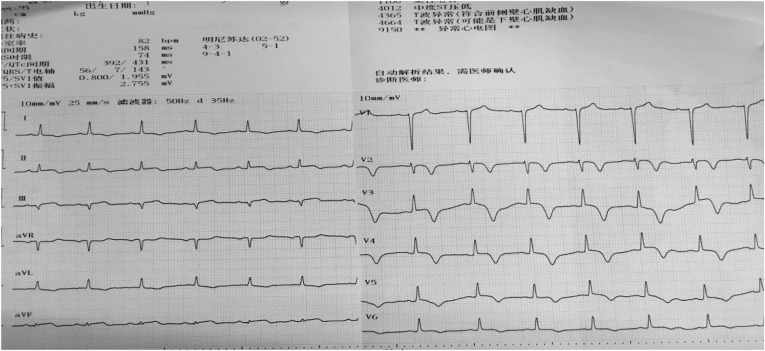
Electrocardiogram on 1 day after operation shows that the elevated ST segments in II, III, aVF, and V4–V6 were down to baseline, and an inverted T wave began to appear in V2–V6.

**Figures 5–7:**
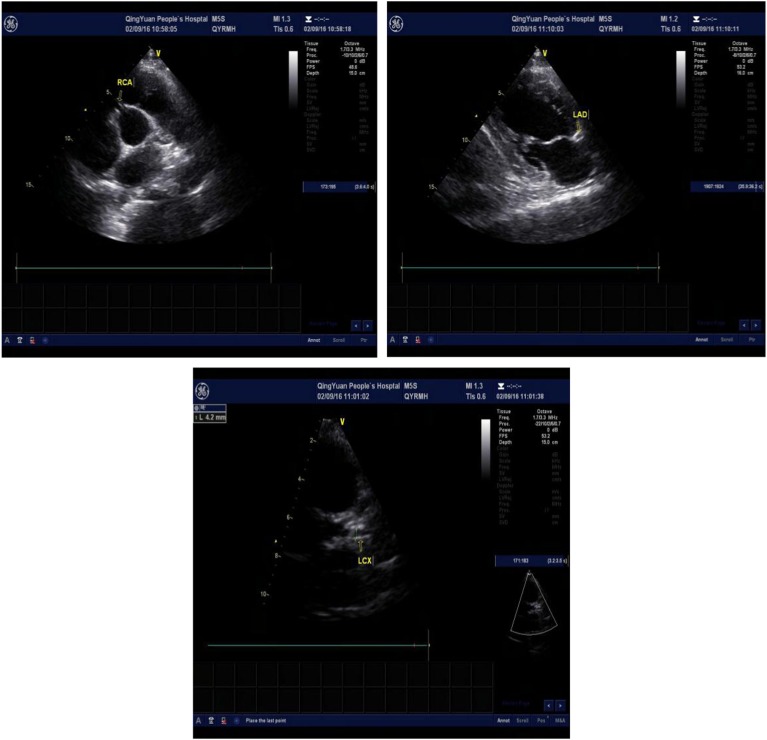
Echocardiography shows the dilated left coronary artery and right coronary artery (RCA) (RCA 4.6 mm, left anterior descending artery (LAD) 6.6 mm, left circumflex artery (LCX) 4.2 mm).

**Figures 8–9:**
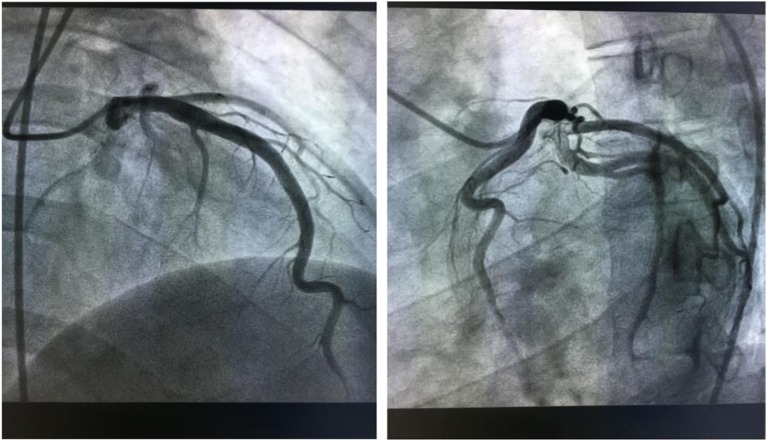
Coronary arteriography on 14th day after the treatment shows barely any blockage caused by thrombi in the anterior descending branch and no stricture in the coronary artery.

One day after the operation, his postoperative electrocardiogram showed that the elevated ST segments in II, III, aVF, and V4–V6 were down to baseline, and an inverted T wave began to appear in V2–V6 (Fig 4). An echocardiography performed 3 days after admission showed a left ventricular ejection fraction of 48.2%, enlarged bilateral atrium, dilated left ventricle, segmental weak pulse of left ventricular wall, mild regurgitation of bicuspid valve or of tricuspid valve, and the estranged left ventricular cells (Figs 5–7). Laboratory tests on the same day showed a higher troponin I of 31.833 ug/L, lower myoglobin of 27.9 ug/L, creatine kinase-MB of 21 U/L, creatine kinase of 74 U/L, and lactate dehydrogenase of 489 U/L. After 4 days, the serum laboratory results showed the following results: troponin I, 6.912 ug/L; myoglobin, 29.5 ug/L; creatine kinase-MB, 17 U/L; creatine kinase, 36 U/L; lactate dehydrogenase, 330 U/L; cholesterol, 2.86 mmol/L; low-density lipoprotein cholesterol, 1.63 mmol/L; triglyceride, 2.28 mmol/L; and high-density lipoprotein cholesterol, 0.57 mmol/L. All the related indexes were decreased. On the 14th day after the operation, the patient had a third examination of coronary arteriography, which revealed that there was barely any blockage caused by thrombi in the anterior descending branch and no stricture in the coronary artery (Figs 8 and 9). The patient was discharged on the 17th day, as his vitals were stable and symptoms disappeared.

## Discussion

The condition of proteinuria, hypoalbuminaemia, and hyperlipidemia, combined with renal biopsy, confirms the diagnosis of nephrotic syndrome.^3^ In addition, the electrocardiography and laboratory results on admission showed that he had acute myocardial infarction. We consider that his acute myocardial infarction probably may have been due to arterial thrombosis, which can be attributed to a hypercoagulable state resulting from the nephrotic syndrome because he had not taken any drugs since he got the disease.^4^

The pathophysiology of myocardial infarction in patients with nephrotic syndrome remains unclear. According to former similar case reports, hyperlipidaemia was deemed to be the most important and independent factor in patients who suffer from acute cardiovascular complications of nephrotic syndrome.^5^ Whether patients with nephrotic syndrome at high-risk condition of cardiovascular diseases or not depend on the level of serum high-density lipoprotein, actually, the level of protective high-density lipoprotein in their blood is usually lower than normal person.^6^ The laboratory results of this case showed familial combined hyperlipidaemia along with a low level of high-density lipoprotein on admission; in addition, as we tested serum lipids for his next of kin, we also testified that five of six of this family members had low-density lipoprotein receptor gene mutation.^7^ However, the block caused by thrombi in the anterior descending branch vanished and hyperlipidaemia was remedied by treatment with statin and antiplatelet drugs, which indicated that hyperlipidaemia probably contributed to the formation of thrombi that accelerated acute coronary syndrome occurrence.^4^

Thromboembolic complications have been frequently reported in patients with long-lasting nephrotic syndrome.^8^ Serious clotting factor disturbances can be observed, such as changes in platelet hyperfunction, increased plasma fibrinogen, abnormalities of the fibrinolytic system, and acquired deficiencies of coagulation inhibitors. However, increased platelet aggregation and antithrombin III deficiency are the most important factors in this hypercoagulable state in nephrotic syndrome.^9^ ^,^ ^10^ To our knowledge, this is the first case of nephrotic syndrome due to membranous nephropathy associated with acute anterior myocardial infarction along with familial hyperlipidaemia.

## Conclusion

The association between nephrotic syndrome and arterial embolisation, especially occlusion of coronary artery, is extremely rare. This case will call our attention to patients with nephrotic syndrome who may have a complication of acute coronary syndrome in addition to venous embolism resulting from familial hyperlipidaemia and hypercoagulable state.
